# Serum periostin as a novel biomarker and therapeutic target in coronary heart disease

**DOI:** 10.3389/fcvm.2025.1556634

**Published:** 2025-12-02

**Authors:** Qian Su, Zhipeng Deng, Lu Li, Shu He, Boxiang Du, Lei Yao

**Affiliations:** 1 Department of Anesthesiology, The Second Affiliated Hospital of Nantong University, Nantong, Jiangsu, China; 2 Department of Epidemiology and Biostatistics, School of Public Health, Nantong University, Nantong, Jiangsu, China

**Keywords:** coronary artery disease (CAD), prognosis, serum periostin (POSTN), therapeutic targets, pathways

## Abstract

**Background:**

Coronary artery disease (CAD) remains a significant global contributor to morbidity and mortality, necessitating a deeper understanding of its molecular mechanisms. This study aimed to identify and analyze differentially expressed genes associated with CAD, assess their functional enrichment, and construct protein-protein interaction (PPI) networks to elucidate the molecular mechanisms underlying the disease.

**Methods:**

Data integration from two Gene Expression Omnibus datasets (GSE66360 and GSE42148) was performed using bioinformatics tools, with batch effects corrected via the R package sva. Differential gene expression analysis was conducted using the limma package in R, enabling identification of genes with statistically significant expression differences between patients with CAD and healthy controls. *POSTN* (periostin) expression was validated through receiver operating characteristic curve analysis. Functional enrichment was assessed using Gene Ontology and Kyoto Encyclopedia of Genes and Genomes analyses via clusterProfiler package, in addition to Gene Set Enrichment Analysis (GSEA) and Gene Set Variation Analysis (GSVA). PPI networks were constructed using GeneMANIA, while regulatory networks were visualized with Cytoscape.

**Results:**

A total of 704 differentially expressed genes were identified, with 476 upregulated and 228 downregulated genes in CAD samples. *POSTN* demonstrated significant differential expression indicating potential diagnostic relevance. Functional enrichment analyses highlighted key biological processes such as regulation of cell junction assembly and wound healing. GSEA highlighted the enrichment of apoptosis-related pathways, while GSVA showed notable differences in epithelial-mesenchymal transition pathways between groups with high and low *POSTN* expression. Immune infiltration analysis revealed distinct immune cell correlations based on *POSTN* expression levels.

**Conclusion:**

This integrated bioinformatics analysis provided valuable insights into the molecular landscape of CAD, highlighting *POSTN* as a potential biomarker for diagnosis and prognosis. Future research is needed to validate these findings in clinical settings and explore therapeutic targets within the identified pathways.

## Introduction

Coronary artery disease (CAD), also known as coronary heart disease, represents one of the most significant health challenges globally. It is the leading cause of mortality worldwide, accountable for approximately 17.8 million deaths annually (1). CAD develops when the coronary arteries, which supply blood to the heart muscle, become narrowed or obstructed due to the accumulation of atherosclerotic plaques, resulting in myocardial ischemia and infarction (2). The prevalence of CAD is concerning, affecting millions of individuals worldwide, with its incidence expected to rise due to increasing rates of risk factors such as obesity, diabetes, and hypertension (3). In the United States, it is estimated that approximately 16.5 million adults have been diagnosed with CAD (4).

The diagnosis and management of CAD encompasses pharmacological therapy, interventional procedures, and surgical interventions (5). However, these approaches are constrained by several limitations, including suboptimal individualized treatment outcomes and high recurrence rates. A gene of particular interest in CAD research is *POSTN* (periostin), a matricellular protein that plays a crucial role in tissue repair and remodeling through interactions with various cell surface receptors and extracellular matrix components (6). It is involved in processes such as cell adhesion, migration, and survival, and is predominantly expressed in the periosteum and periodontal ligament (7). Recent studies have emphasized the significance of *POSTN* in cardiovascular diseases, including its role in cardiac fibrosis and heart failure (8).

Previous research has demonstrated that *POSTN* is upregulated in response to myocardial infarction and contributes to the fibrotic remodeling of the heart, a key pathological feature of CAD (9). Elevated levels of periostin have been detected in the serum of patients with acute myocardial infarction, suggesting its potential as a biomarker for cardiac injury (10). Additionally, *POSTN* has been implicated in the regulation of inflammatory responses in the cardiovascular system, further highlighting its role in the pathogenesis of CAD (11). Despite these findings, the precise molecular mechanisms by which *POSTN* influences CAD remain poorly understood, necessitating further investigation. While an association between *POSTN* and CAD has been suggested in previous studies, no human studies have, to the author's knowledge, been conducted to directly investigate this relationship (8–11).

The use of bioinformatics methods in this study offers a significant advantage in managing large-scale datasets and elucidating complex biological interactions that may not be detectable through traditional experimental approaches (12). These methodologies were employed to gain a comprehensive understanding of gene expression changes and molecular pathways associated with CAD. Specifically, the focus was on the *POSTN* gene, to investigate its role and regulatory mechanisms in CAD, with the potential to yield valuable insights for the development of novel diagnostic and therapeutic strategies. In summary, the integration of bioinformatics tools enabled the analysis of CAD-related differentially expressed genes (DEGs) and their functional implications, with a particular emphasis on the *POSTN* gene. The findings from this study may enhance the understanding of the molecular mechanisms underlying CAD and help identify new targets for clinical intervention.

## Methods

### Acquisition and screening of disease gene targets

Datasets related to CAD were retrieved from the Gene expression Omnibus (GEO) database using the R package. Specifically, GSE66360 and GSE42148 were selected for analysis, with details provided in Supplementary Table S1. The GSE66360 dataset utilized the GPL570 chip platform and comprised of 49 CAD samples and 50 control samples. The GSE42148 dataset, based on the GPL13607 chip platform, comprised 13 CAD samples and 11 control samples. Subsequently, the R package limma was used to standardize the Combined GEO datasets, annotate probes, and normalize the data. To evaluate the effectiveness of batch effect removal, Principal Component Analysis (PCA) was conducted on the expression matrix following batch effect correction.

### Differentially expressed genes associated with coronary artery disease

DEGs were analyzed using the R package ggplot 2. Differential expression was determined based on a threshold of |logFC| > 0.58 and an adjusted *p*-value (adj.*p*) < 0.05. Genes meeting the criteria of logFC > 0.58 and adj.*p* < 0.05 were classified as upregulated DEGs, while those with logFC < −0.58 and adj.*p* < 0.05 were categorized as downregulated DEGs. The Benjamini-Hochberg (BH) method was applied for *p*-value correction. Additionally, CAD samples from the combined GEO datasets were stratified into high-expression and low-expression groups based on the median expression value of the *POSTN* gene.

### Gene target enrichment analysis

Gene Ontology (GO) and Kyoto Encyclopedia of Genes and Genomes (KEGG) enrichment analysis were performed using the R package Cluster Profiler (version 4.3.0; https://www.gsea-msigdb.org/gsea/index.jsp) on DEGs identified through differential expression analysis between high and low expression groups of CAD samples from the integrated GEO datasets (Combined Datasets). DEGs were filtered using an adjusted *p*-value (adj.*p*) threshold of <0.05, with *p*-value correction performed using the Benjamini-Hochberg (BH) method.

In this study, CAD gene samples from the Combined GEO datasets were ranked according to logFC values. The parameters used in GSEA were as follows: a random seed set to 2020, 1,000 computations, a minimum gene set size of 10, and a maximum gene set size of 500. The Molecular Signatures Database (MSigDB) was accessed (https://www.gsea-msigdb.org/gsea/msigdb), and the GMT file “All Canonical Pathways” (3,050) was used for GSEA (12). A threshold of adj.*p* < 0.05 and a false discovery rate (FDR) value (q value) < 0.25 were considered statistically significant. *P*-value correction was performed using the Benjamini-Hochberg (BH) method.

To evaluate differential pathway enrichment among samples, the c2.cp.v2023.2.Hs. symbols GMT gene set from MSigDB was used. GSVA was performed on the integrated GEO dataset using the R package GSVA (version 1.50.0) to assess differences in functional enrichment between the high and low *POSTN* expression groups. GSVA results were considered significant if the *p*-value was <0.05.

### Protein-protein interaction (PPI) network

The GeneMANIA database (https://genemania.org/) was used to generate hypotheses regarding gene function, analyze gene lists, and prioritize genes for further functional analysis. Using the GeneMANIA online platform, a PPI network was constructed, and core protein targets were identified based on criteria that required both node degree and median centrality values to exceed their respective average values.

### Upstream and downstream regulatory relationships of *POSTN* in cells

Transcription factors (TFs) associated with target genes were predicted via an established TF Regulatory Network via the ChIPBase database (http://rna.sysu.edu.cn/chipbase/). These transcription factors regulate gene expression by interacting with DDB2 at the post-transcriptional stage. The ChIPBase database was utilized to retrieve TFs, and analyze their regulation of the *POSTN* gene. Cytoscape software was employed to visualize the mRNA-TF Regulatory Network.

Additionally, MicroRNAs (miRNAs) play an important regulatory role in biological development and evolution by regulating multiple target genes. Conversely, a single target gene can be regulated by multiple miRNAs. The relationship between *POSTN* and microRNAs was analyzed using the starBase database (https://rnasysu.com/encori/) to identify microRNAs associated with *POSTN*.

Comparative Toxicogenomics Database (https://ctdbase.org/) was used to predict the direct and indirect drug targets of the *POSTN* gene. This facilitated the exploration of the interactions between *POSTN* and various drugs.

### Molecular docking

Following the construction of the mRNA Drug Regulatory Network, potential small molecule compounds capable of directly or indirectly targeting *POSTN* were identified.

The AutoDock Vina program on the CB-Dock2 platform (https://cadd.labshare.cn/cb-dock2/php/index.php) was utilized to conduct blind docking and visualize the interaction between *POSTN* and small molecule compounds. The docking strength was evaluated using the Vina Score, which reflects the binding affinity. A Vina Score >−4 Kcal/mol, indicates negligible or no binding affinity, while a score between −7 Kcal/mol and −4 Kcal/mol represents moderate binding affinity. A Vina Score <−7 Kcal/mol is considered indicative of strong binding affinity.

### Immune infiltration analysis

The proportions of the 22 types of immune cells in samples were determined using the CIBERSORT algorithm. Correlation analysis between the *POSTN* gene and the immune cells was performed and visualized using the R package ggplot 2 (Version 3.4), available at https://ggplot2.tidyverse.org https://github.com/tidyverse/ggplot2.

### Statistical analysis

Data processing and analysis were conducted using R software (Version 4.3.0). Receiver operating characteristics (ROC) curve analysis was used to evaluate the diagnosis and prognostic capabilities of *POSTN*. Unless otherwise specified, statistical significance of normally distributed variables was assessed using independent Student's *t*-test for comparisons of continuous variables between two groups. For non-normally distributed variables, the Mann–Whitney U Test method (Wilcoxon Rank Sum Test) was used. Kruskal–Wallis test was used for comparison among three or more groups. Spearman's correlation analysis was used to determine the correlation coefficient between different molecules. All *p*-values were two-sided with a significance threshold of <0.05.

## Results

### Expression of *POSTN* in CAD samples

**Figure 1:**
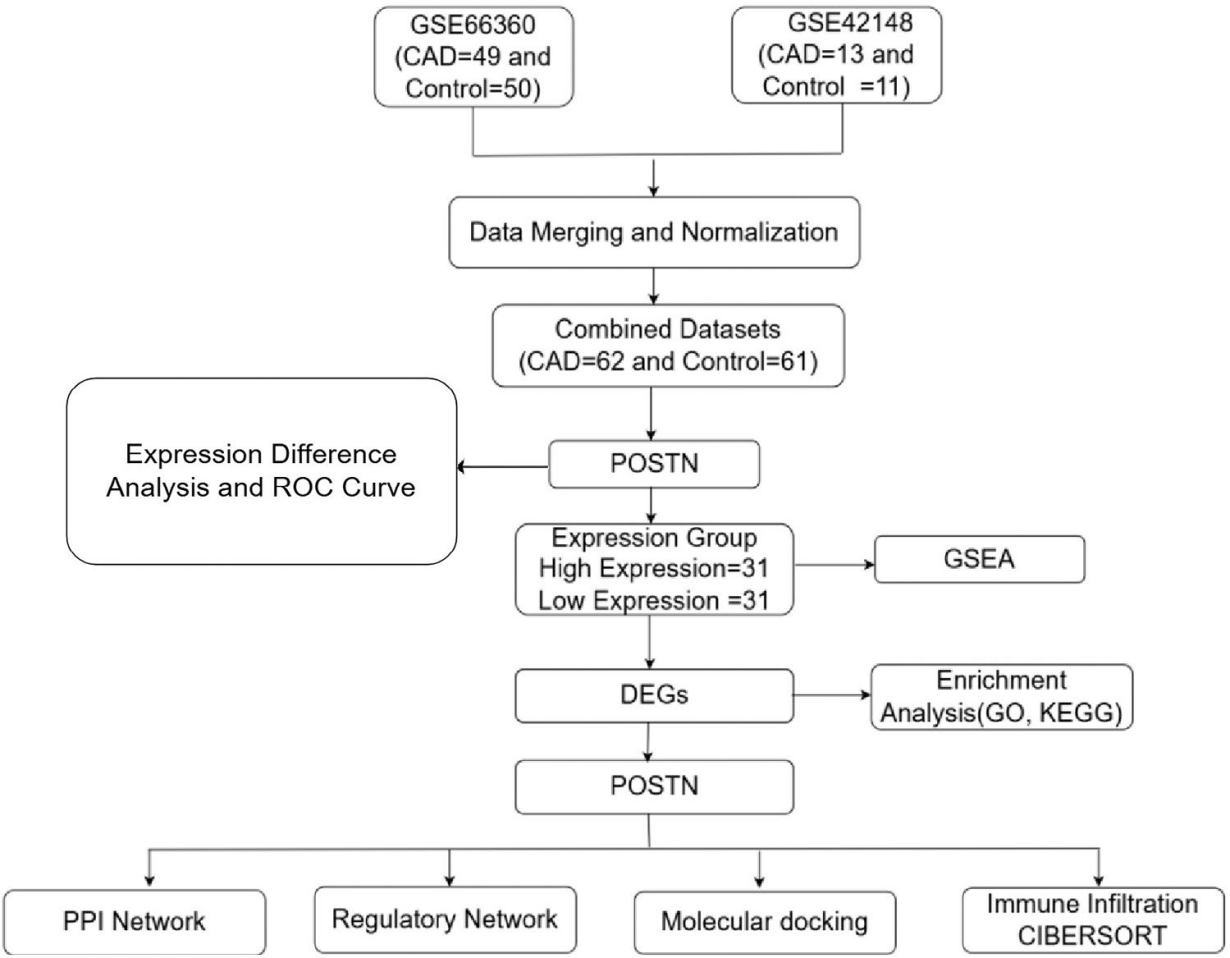
Flow chart for the comprehensive analysis of *POSTN.* CAD, Coronary artery disease; DEGs, Differentially expressed genes; ROC, Receiver operating characteristic; GSEA, Gene set enrichment analysis; GO, Gene ontology; KEGG, Kyoto encyclopedia of genes and genomes; PPI, Protein-protein interaction; TF, Transcription factor; CIBERSORT, Cell-type identification by estimating relative subsets of RNA transcripts.

**Figure 2:**
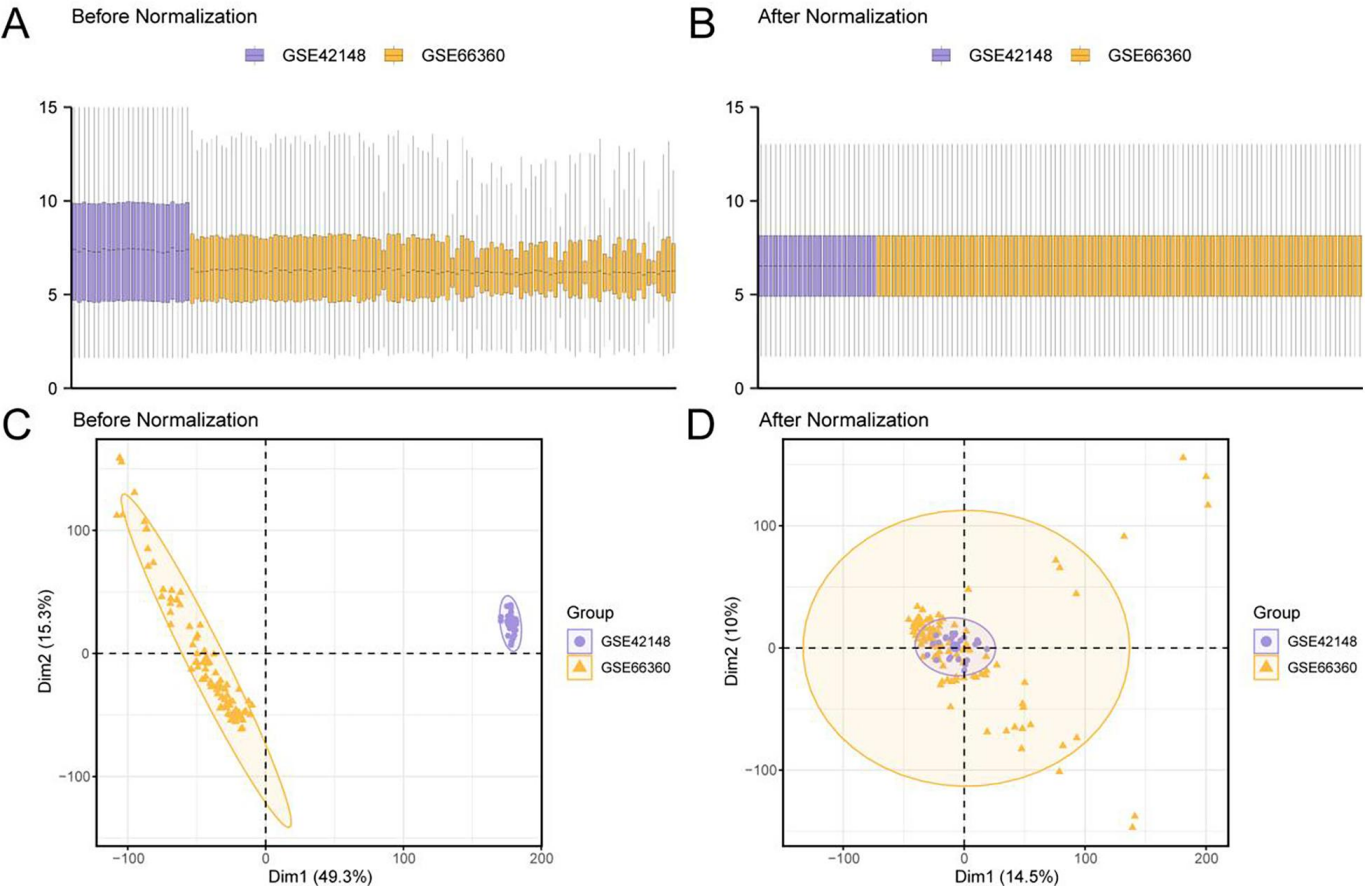
Batch effects removal of GSE66360 and GSE42148. **(A)** Box plot of Combined GEO Datasets distribution before batch removal. **(B)** Post-batch integrated GEO Datasets (Combined Datasets) distribution boxplots. **(C)** PCA plot of integrated GEO Datasets (Combined Datasets) before debatching. **(D)** PCA plot of integrated GEO Datasets (Combined Datasets) after debatching. PCA, Principal component analysis; CAD, Coronary artery disease. The coronary artery disease (CAD) dataset GSE66360 is shown in purple, and the coronary artery disease (CAD) dataset GSE42148 is shown in yellow.

Two datasets, GSE66360 and GSE42148, were obtained from the GEO database for analysis. The combined datasets included 62 CAD samples and 61 control samples (Figure 1). *POSTN* expression was found to be higher in CAD samples compared to control samples. (Figure 2).

The results from the distribution box plot (Figures 2A,B) and the PCA plot (Figures 2C,D) demonstrated that the batch effect in the CAD dataset was effectively removed after batch correction.

A total of 704 DEGs were identified from the combined datasets including 476 upregulated genes and 228 downregulated genes. Differential expression results indicated a highly significant difference in *POSTN* expression between CAD samples and control samples (*p* < 0.001). The ROC curve analysis indicated that *POSTN* expression exhibited moderate diagnostic accuracy across different groups (AUC = 0.712, 95%CI: 0.621–0.803, Table 1).

**Table 1:** Diagnostic performance of POSTN expression for different groups.

Variable	Cut-off value	Sensitivity	Specificity	Youden's index	AUC	95%CI
POSTN	4.058	0.435	0.934	0.370	0.712	0.621–0.803

### Differentially expressed genes related to CAD in high and low *POSTN* expression groups

Analysis of the integrated GEO Datasets (Combined Datasets) for CAD identified 233 DEGs, comprising 152 upregulated genes and 81 downregulated genes (Supplementary Table S1).

**Figure 3:**
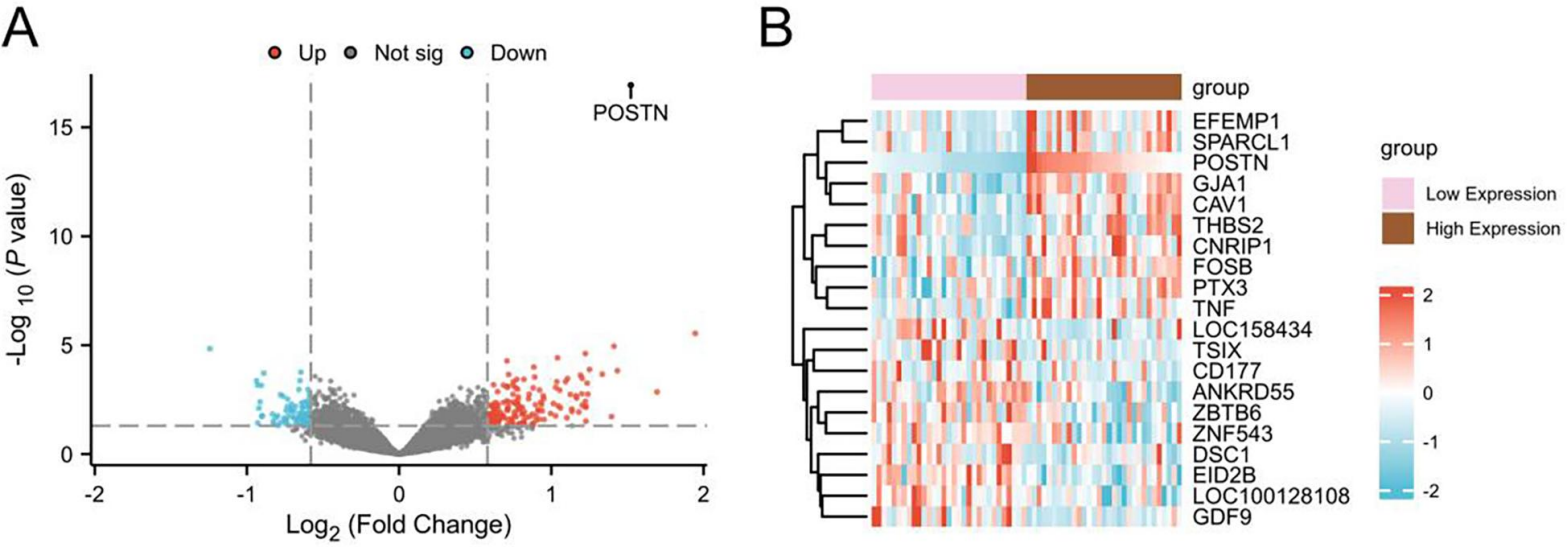
Differential gene expression analysis. **(A)** Volcano plot of differentially expressed genes analysis between High Expression group and Low Expression group in coronary artery disease (CAD) samples of Combined GEO Datasets. **(B)** Heat map of logFC-ranked TOP10 up-regulated and TOP10 down-regulated differentially expressed genes (DEGs) in coronary artery disease (CAD) samples from integrated GEO Datasets (Combined Datasets). CAD, Coronary artery disease; DEGs, Differentially expressed genes. In the heat map group, the brown is the High Expression group, and the pink is the Low Expression group. In the heat map, red represents high expression and blue represents low expression.

A volcano plot was generated to visualize these DEGs, illustrating the results of the differential expression analysis (Figure 3A). A heatmap was used to depict the expression profiles of the 233 DEGs in the low risk and high risk groups. In the heatmap, red represents the high risk group, blue represents the low risk group, and the intensity of the color corresponds to the level of gene regulation. Upregulated DEGs are shown in red and downregulated DEGs are shown in blue. The volcano plot corroborates these findings, with downregulated genes highlighted in the blue region and the upregulated genes highlighted in the red region (Figure 3B).

### GO and KEGG enrichment analysis

**Figure 4:**
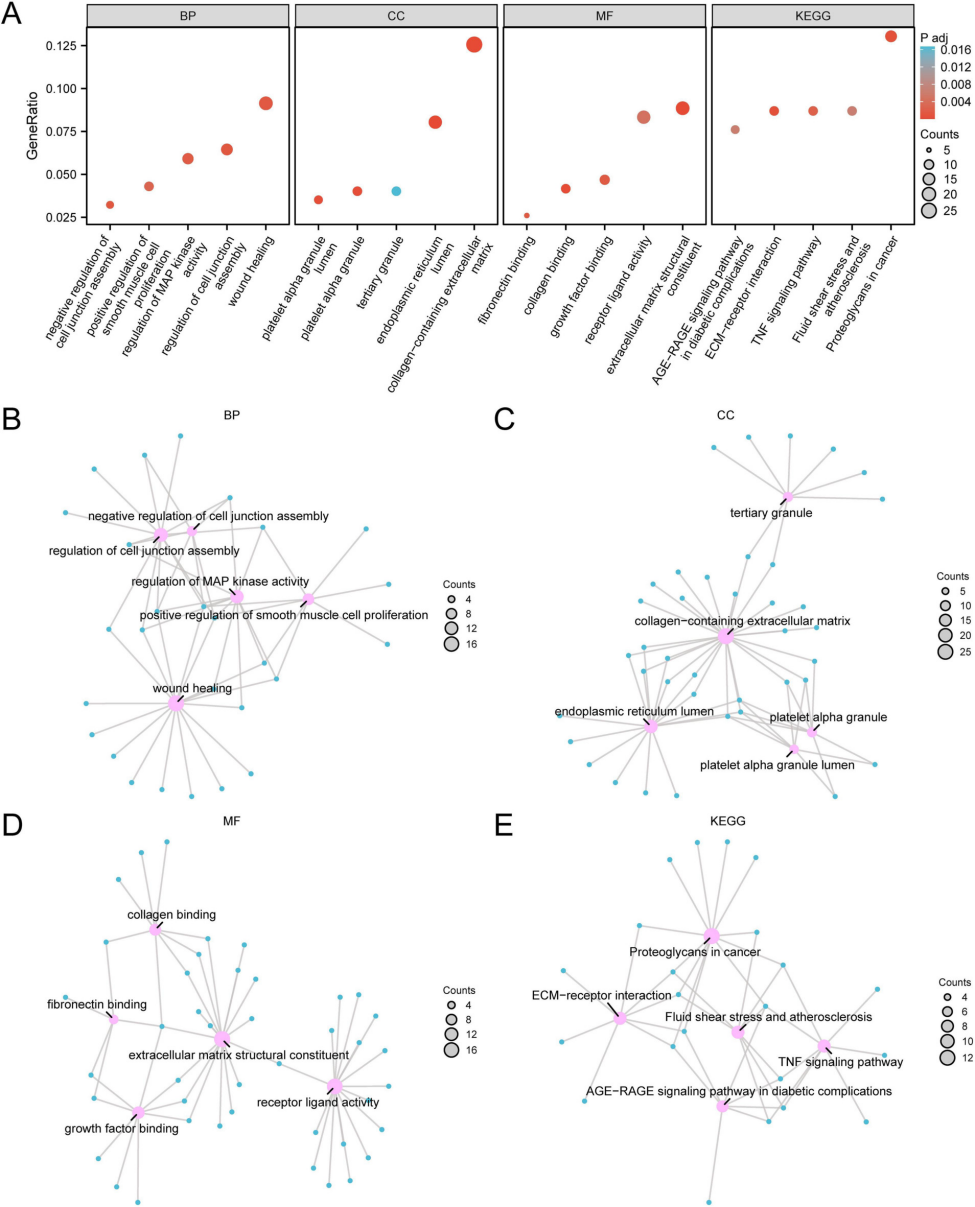
GO and KEGG enrichment analysis for DEGs. **(A)** GO, Gene ontology; and pathway (KEGG) enrichment analysis results of differentially expressed genes (DEGs) bubble plot **(A)** showing: BP, Biological process; CC, Cellular component; MF, Molecular function; KEGG, biological Pathway. GO terms and KEGG terms are shown on the abscissa. **(B–E)** GO, Gene ontology; pathway (KEGG) enrichment analysis results network diagram of differentially expressed genes (DEGs): BP **(C)**, CC **(D)**, MF **(E)** and KEGG **(F)**. Pink nodes represent items, blue nodes represent molecules, and lines represent the relationship between items and molecules. DEGs, Differentially expressed genes; GO, Gene ontology; KEGG, Kyoto encyclopedia of genes and genomes; BP, Biological process; CC, Cellular component; MF, Molecular function. The bubble size in the bubble plot represents the number of genes, and the color of the bubble represents the size of the adj. *P*-value, the reder the color, the smaller the adj. *P*-value, and the bluer the color, the larger the adj. *P*-value. The screening criteria for gene ontology (GO) and pathway (KEGG) enrichment analysis was adj.*p* < 0.05.

The GO analysis indicated that the 233 DEGs are primarily enriched in pathways related to the extracellular matrix and wound healing (Supplementary Table S2, Figure 4). These findings may provide insights into the mechanisms underlying ventricular remodeling in patients with CAD and offer potential clues for identifying therapeutic targets.

The KEGG analysis identified significant alterations in pathways associated with proteoglycans in cancer, fluid shear stress, and atherosclerosis (Supplementary Table S2). Consistent with the RNA-sequencing results, an upregulation of metabolites in glycine, serine, and threonine metabolism was observed, along with the downstream pathways involving glycine, cystine, and l -cystathionine.

### Gene set enrichment analysis (GSEA) of CAD

**Figure 5:**
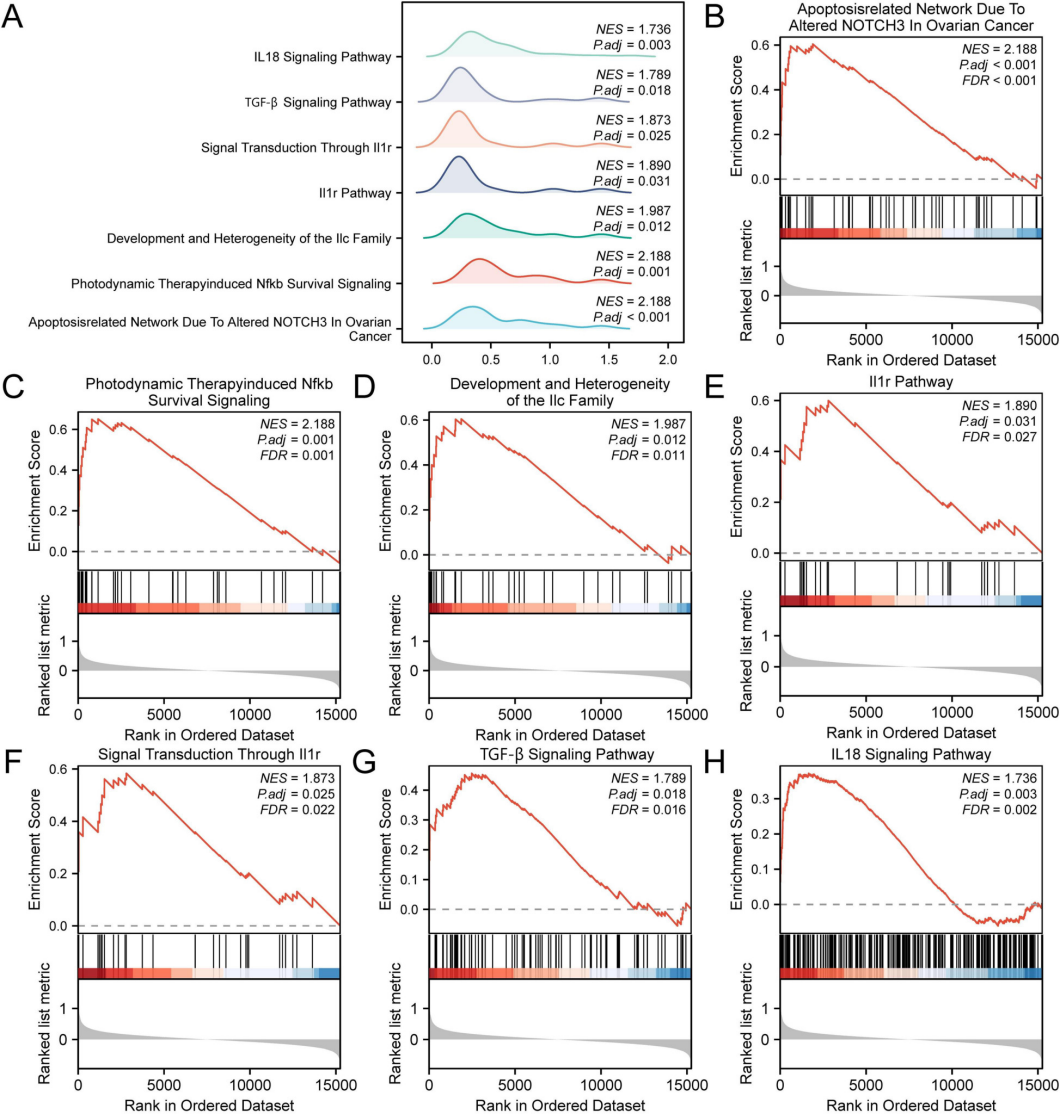
GSEA for expression group. Gene set enrichment analysis (GSEA) of coronary artery disease (CAD) samples in the Combined GEO Datasets shows 7 biological function mountain plots **(A)**. Gene set enrichment analysis (GSEA) showed that all genes were significantly enriched in the Apoptosis related Network Due To Altered NOTCH3 In Ovarian Cancer **(B)**, Photodynamic Therapy induced NF-κB Survival Signaling **(C)**, Development And Heterogeneity Of The Ilc Family **(D)**, Il1r Pathway **(E)**, Signal Transduction Through Il1r **(F)**, TGF-β Signaling Pathway **(G)** and IL18 Signaling Pathway **(H)**. CAD, Coronary artery disease; GSEA, Gene set enrichment analysis. *p* < 0.05 and FDR value (*q* value) < 0.25 were considered statistically significant. The *p* value correction method was Benjamini-Hochberg (BH).

The GSEA results indicated that all genes in the combined datasets were significantly enriched in the apoptosis-related network and the nuclear factor kappa-light-chain-enhancer of activated B cells (NF-κB) signaling pathways (Supplementary Table S3, Figure 5). These findings suggest that a deficiency in *POSTN* may lead to the activation of apoptosis-related networks and the NF-κB signaling pathways.

### Gene set variation analysis (GSVA) of CAD

The GSVA results indicated that epithelial-mesenchymal transition (EMT) was statistically significant in both the low expression and high expression groups of *POSTN* (*p* < 0.001, Supplementary Table S4). EMT signatures were enriched in the high expression group (*p* < 0.001). Additionally, significant differences were observed in the low expression and high expression groups for glycolysis, angiogenesis, and TGF-β signaling pathways (*p* < 0.05).

### Construction of PPI network

PPI analysis identified 20 genes that interact with *POSTN* at the protein level (Supplementary Table S2, Figure S1). These findings suggested that *POSTN* may play an important role in the stromal inflammatory environment associated with CAD and may be a potential biomarker or target for CAD.

### Construction of regulatory networks

In previous studies, miR-144-3p and miR-30e-3p have been identified as significantly associated with poor prognosis, consistent with the principle of negative regulation of their target genes (13). In this study, 9 miRNAs were found to potentially regulate the expression of *POSTN* (Supplementary Figure S2). These miRNAs include miR-518d-3p, miR-4465, miR-26b-5p, miR-221-3p, miR-302a-3p, miR-302b-3p, miR-518f-3p, miR-518c-3p, and miR-518a-3p. Several of these miRNAs have been linked to critical biological processes and disease outcomes: miR-518, miR-44650, and miR-26b-5p are associated with apoptosis of tumor cells (14, 15). MiR-26b-5p demonstrated a capacity to downregulate the expression of inflammatory factors, alleviating cerebral ischemia-reperfusion injury in rat models, suggestive of its protective role against cerebral infarction (16). MiR-221-3p suppresses angiogenesis in endothelial cells by targeting HIF-1α, with evidence showing that blocking miR-221-3p enhances cardiac function in mice with transverse aortic constriction (TAC)-induced heart failure (17). Inhibition of miR-302a-3p has been shown to mitigate myocardial ischemia-reperfusion injury (18).

### Molecular docking

**Figure 6:**
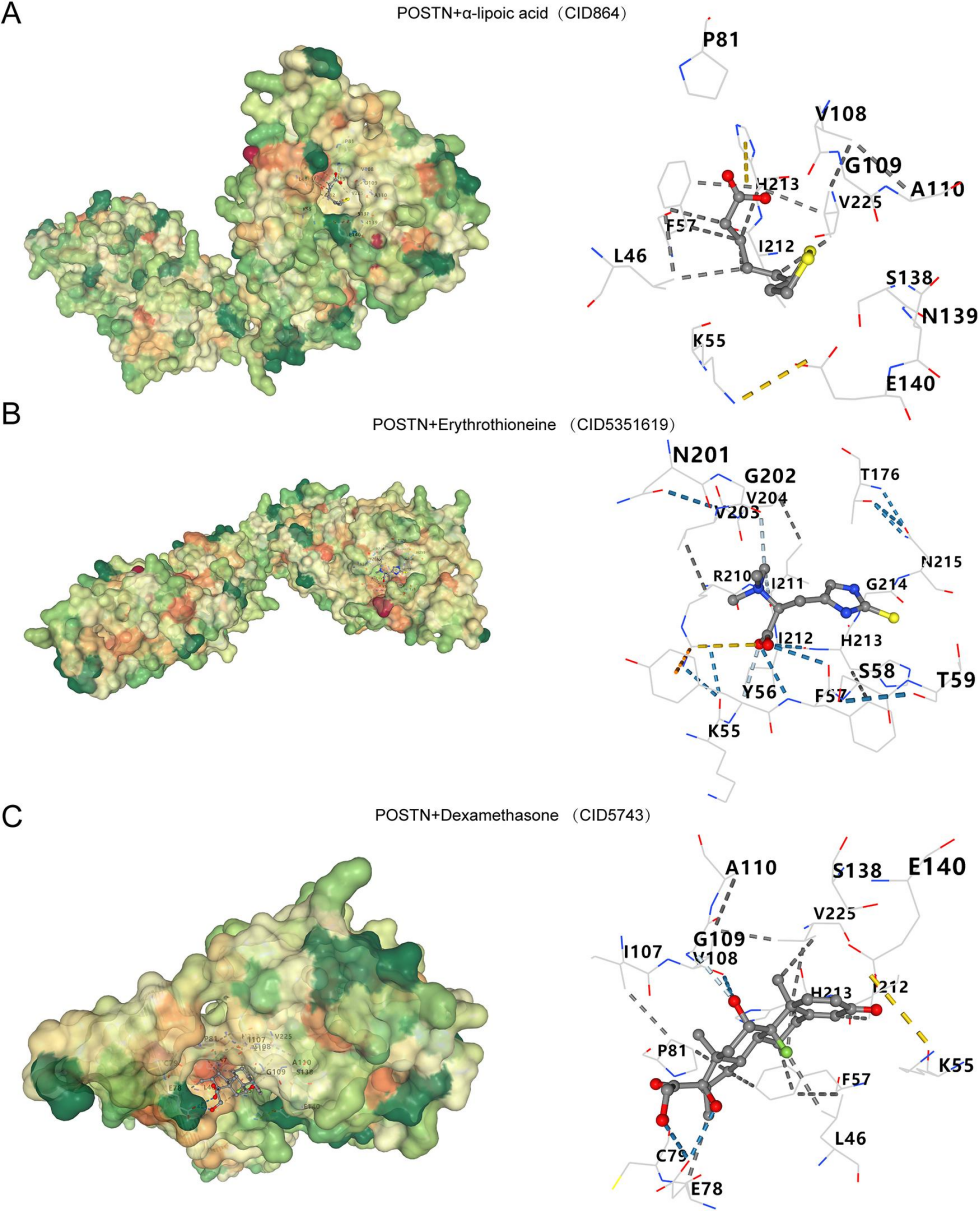
Molecular docking. Gene *POSTN* with α-lipoic acid **(A)**. Gene *POSTN* with Erythrothioneine **(B)**. Gene *POSTN* with Dexamethasone **(C)**. The color of the protein surface changes from green, orange, and red to indicate that the amino acid properties change from hydrophilic to hydrophobic. The blue dotted line—is a hydrogen bond, the light blue dotted line is—a weak hydrogen bond, the gray dotted line is—hydrophobic force, the yellow dotted line is—ionic bond, and the orange dotted line is—cation-π interaction.

**Figure 7:**
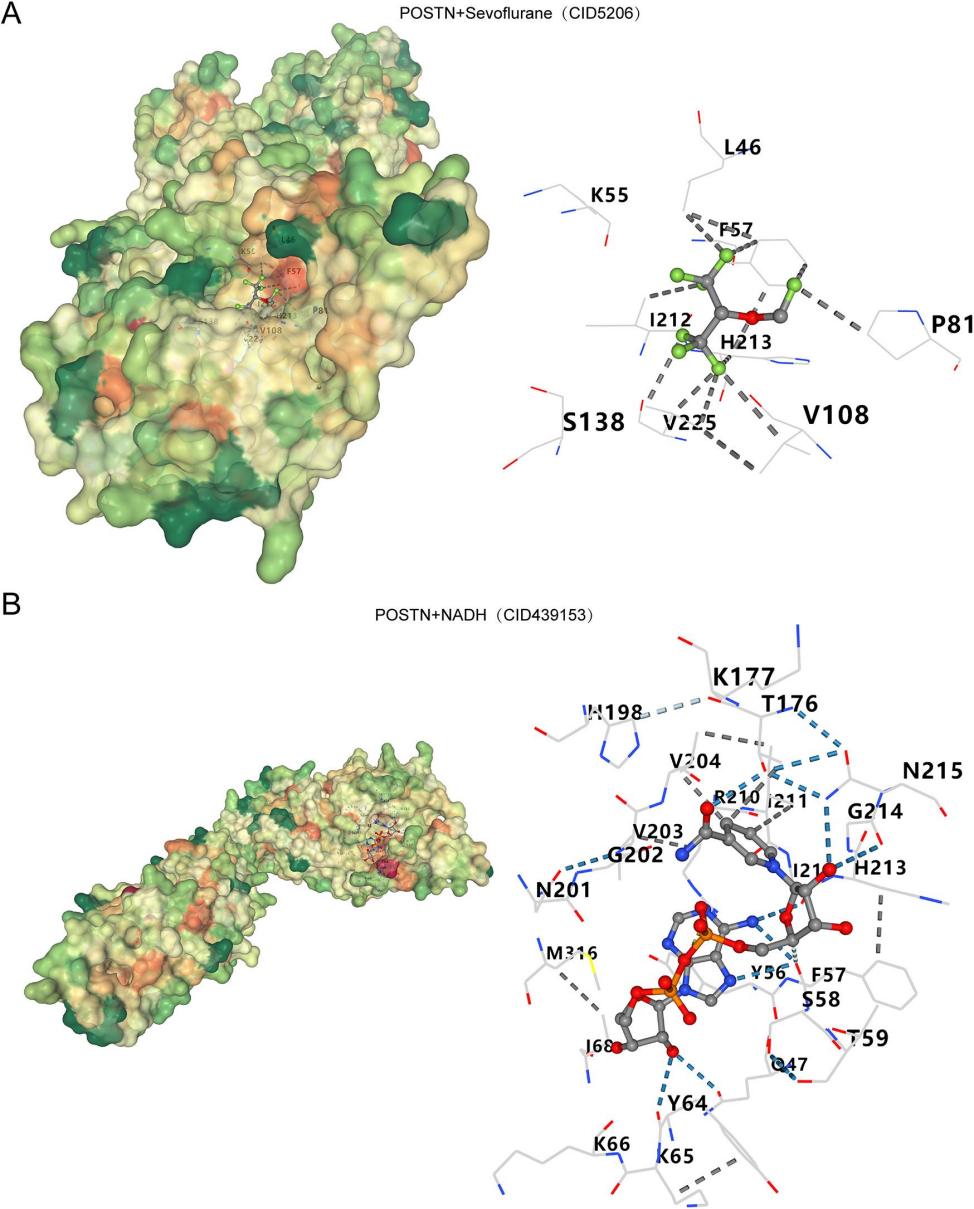
Global map and interaction force map of the gene *POSTN* with sevoflurane **(A)** and NADH **(B)** the color of the protein surface changes from green, orange, and red to indicate that the amino acid properties change from hydrophilic to hydrophobic. The blue dotted line—hydrogen bond, the light blue dotted line—weak hydrogen bond, and the gray dotted line—hydrophobic force.

Six drugs or molecular compounds potentially interacting with *POSTN* were identified: α-lipoic acid, ergothioneine, dexamethasone, sevoflurane, and NADH (Supplementary Table S5). Molecular docking was performed using the CB-Dock 2 platform (https://cadd.labshare.cn/cb-dock2/php/index.php). The results of the docking analysis are provided in Supplementary Table S5, with visualizations provided in Figures 6, 7. The binding affinities between *POSTN* and various small molecules were ranked in descending order based on their docking energies: NADH, dexamethasone, erythrothioneine, α-lipoic acid, and sevoflurane. NADH exhibited the strongest binding affinity with *POSTN*, characterized by numerous dark blue hydrogen bonds, indicating robust molecular interactions.

### Immune infiltration analysis of high and low *POSTN* expression groups

**Figure 8:**
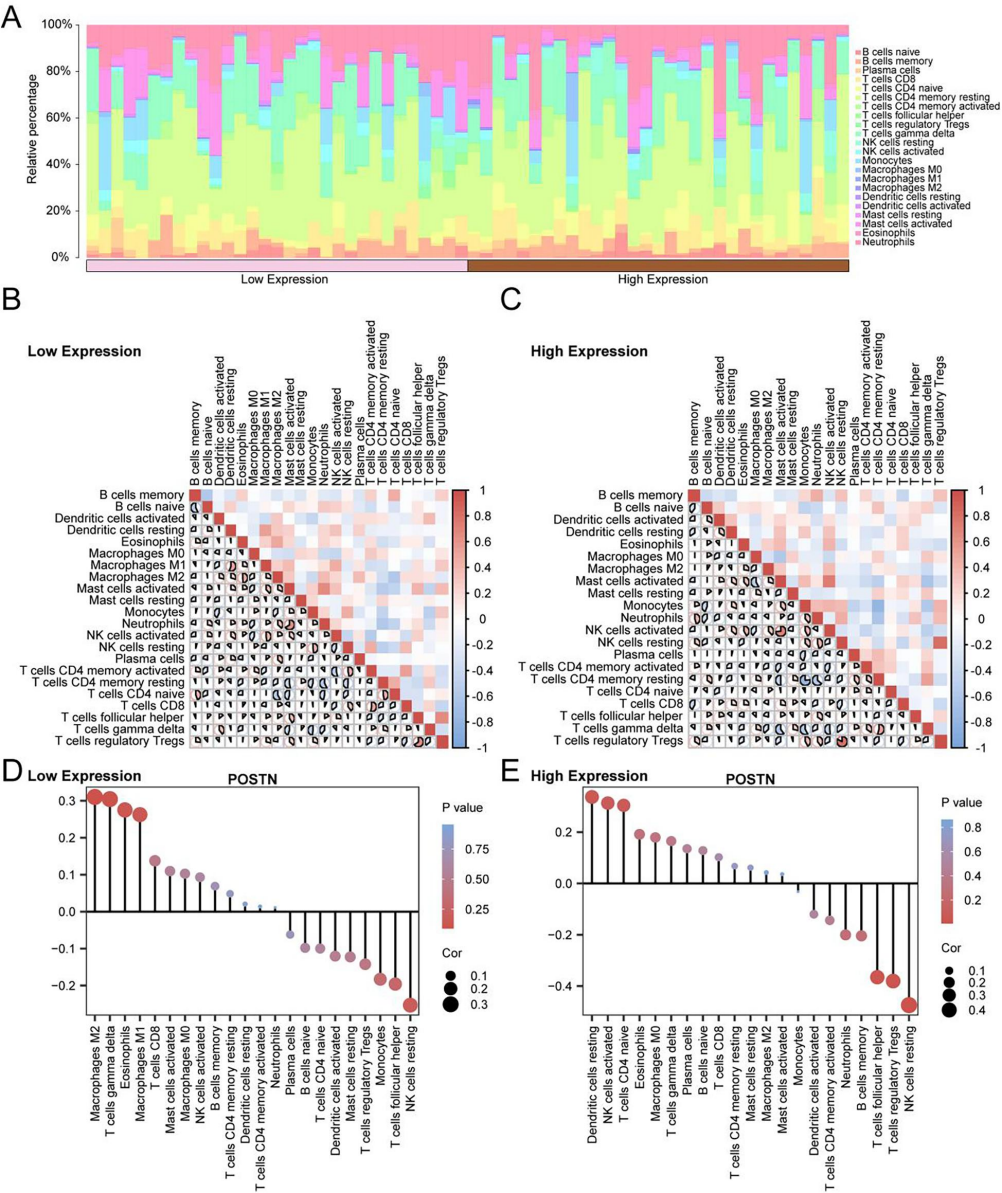
Immune infiltration analysis by CIBERSORT algorithm Bar chart of the proportion of immune cells in coronary artery disease (CAD) samples **(A)**. **(B,C)** Correlation heat map of immune cells in Low Expression group **(B)** and High Expression group **(C)** of coronary artery disease (CAD) samples. **(D,E)** Bubble plot of correlation between immune cell infiltration abundance and gene *POSTN* in Low Expression group **(D)** and High Expression group **(E)** of coronary artery disease (CAD) samples. CAD, Coronary artery disease. The absolute value of correlation coefficient (*r* value) below 0.3 was weak or no correlation, between 0.3 and 0.5 was weak correlation, between 0.5 and 0.8 was moderate correlation, and between 0.8 and 1 was strong correlation. Brown is the High Expression group, pink is the Low Expression group. In the heat map, red is the positive correlation, and blue is the negative correlation. The depth of the color represents the strength of the correlation.

The immune infiltration analysis identified 22 immune cell types in CAD samples. These included naïveB cells, activated B cells, plasmacytoid dendritic cells, activated CD8^+^ T cells, naive CD4^+^ T cells, activated CD4^+^ T cells, central memory CD4^+^ T cells, T follicular helper cells (Tfh), regulatory T cells (Treg), Gamma-delta T cells, resting NK cells, natural killer cells, macrophages (M0, M1, and M2), plasmacytoid dendritic cells, activated dendritic cells, mast cells, activated mast cells, eosinophils, and neutrophils (Figure 8A).

Activated mast cells and neutrophils exhibited the strongest positive correlation in the low *POSTN* expression group (*r* = 0.656, *p* < 0.05, Figures 8B,C). In the high expression group, the strongest positive correlation was observed between regulatory T cell (Treg) and resting NK cells (*r* = 0.83, *p* < 0.05). The correlation between *POSTN* and immune cell infiltration abundance was visualized using a correlation lollipop plot (Figures 8D,E). In the low expression group, *POSTN* had the strongest positive correlation with M2 macrophages (*r* = 0.31). In the high expression group, *POSTN* had the strongest negative correlation with resting NK cells (*r* = −0.474, *p* < 0.05).

## Discussion

CAD accounts for more than 9 million deaths annually, making it the leading cause of mortality worldwide (19). Current treatment modalities, including drug therapy, coronary artery bypass graft (CABG), and percutaneous coronary intervention (PCI) are effective but not without limitations. They carry risks such as gastric bleeding due to anticoagulant therapy, pulmonary embolism and stroke caused by CABG, and left atrial puncture caused by PCI. Despite the high global prevalence, morbidity, and mortality associated with CAD, there is a paucity of studies forecasting its future trends and focusing on developing safer therapeutic approaches.

This study highlights that *POSTN* is highly expressed in CAD samples, aligning with previous research and emphasizing its potential as a promising biomarker for predicting the CAD prognosis (20). Additionally, the investigation into immune pathways associated with *POSTN* provides potential targets for immunotherapy in CAD. In the diagnosis and assessment of cardiovascular disease (CAD), the multidimensional evaluation system of biomarkers is an important research direction. *POSTN* (Periostin), as an emerging biomarker, shows potential to complement existing CAD biomarkers such as high-sensitivity C-reactive protein (hs-CRP) and cardiac troponin I (cTnI), thereby contributing to the establishment of a multidimensional biomarker evaluation system encompassing “structure—inflammation—damage”. These findings align with previous studies that have identified DEGs in various subsets of patients diagnosed with cardiovascular disease, further supporting the feasibility of tailoring treatment strategies to individual patient profiles (21).

This study identified DEGs in CAD that are significantly associated with negative regulation of cell junction assembly, wound healing, and MAP kinase activity regulation, as well as collagen binding and extracellular matrix (ECM) components. Endothelial cells rely on tight junctions to regulate vascular permeability and integrity. Disruption of these junctions exacerbates vascular permeability, promotes inflammation, and accelerates CAD progression. These observations are consistent with the findings of previous studies, which highlighted the importance of these pathways in maintaining cellular integrity and facilitating tissue repair processes, both of which are often compromised in CAD (22–24).

The investigation also explored the potential involvement of *POSTN* in cardiac muscular repair. The endogenous pathway in CAD involves cellular stressors, including oxidative stress and DNA damage, that trigger the release of cytochrome c and other pro-apoptotic factors from mitochondria (25). These factors subsequently bind to apoptotic protease activating factor 1 (Apaf-1), forming apoptosomes, leading to the activation of caspase-9. This cascade further stimulates downstream effectors such as caspase-3; ultimately inducing apoptosis and impacting cell survival. The exogenous apoptotic pathway is initiated through the activation of cell surface death receptors by external apoptotic signals, such as inflammatory factors. This activation recruits proteins that activate caspase-8, which can directly or indirectly activate downstream proteases, ultimately resulting in apoptosis (26).

This study identified a potential role of the NF-κB pathway in the inflammatory environment of CAD. In CAD, inflammatory factors activate the NF-κB pathway, resulting in the degradation of its inhibitory proteins. This allows NF-κB to translocate into the nucleus. This process initiates the transcription of anti-apoptotic genes such as Bcl-2 and Bcl-xL. These genes suppress apoptosis signals and support cell survival. Conversely, NF-κB regulates genes involved in glycolysis and fatty acid metabolism, prompting cells to adopt glycolysis during myocardial ischemia and hypoxia in CAD. This supports short-term cell survival but may lead to long-term metabolic issues (27, 28).

The findings of this study are supported by GSEA, which indicated significant enrichment in apoptosis-related networks and NF-kB survival signaling pathways. These pathways are crucial in the inflammatory response and cell survival mechanisms within the context of CAD (29). This study aligns with prior research focusing on the role of monocytes and macrophages in progression of CAD, while also highlighting the potential role of NK cells in the inflammatory microenvironment (30, 31). Emerging evidence underscores the significance of diverse immune cell types in CAD such as T cells, B cells, macrophages, and dendritic cells in the development of atherosclerotic plaques. Multiplex immunohistochemistry analyses have shown increased infiltration of CD3^+^ T cells, CD20^+^ B cells, CD68^+^ macrophages, and CD15^+^ neutrophils within the intima of diseased coronary arteries compared to normal arteries, indicative of an active immune response in atherosclerosis (32).

Cytokines and chemokines further contribute to the inflammatory landscape in CAD. For instance, serum levels of monokine induced by gamma interferon (MIG/CXCL9) have been associated with the severity of CAD, suggesting its potential as a biomarker for disease progression (33). Additionally, the overexpression of interleukin-1 receptor type II (IL1R2) in peripheral blood mononuclear cells from patients with CAD highlights the critical involvement of inflammatory pathways in CAD (34).

High-dimensional single-cell analyses have provided deeper insights into the peripheral immune signatures associated with CAD. For instance, Cytometry by Time-of-Flight (CyTOF) analysis has identified specific peripheral immune cell subsets that correlate with the severity of coronary atherosclerosis, highlighting their potential as minimally invasive biomarkers for disease monitoring (35). Additionally, the decreased number of circulating plasmacytoid dendritic cells in patients with CAD suggests a potential role in plaque progression and instability (36).

The immune infiltration analysis conducted in this study provided distinct insights into cell interactions in high and low *POSTN* expression groups. Specifically, in the low *POSTN* expression group, activated mast cells and neutrophils showed the strongest positive correlation, whereas in the high *POSTN* expression group, regulatory T cells (Tregs) and resting NK cells exhibited the strongest positive correlation. Furthermore, in the low *POSTN* expression group, *POSTN* showed a positive correlation with M2 macrophages, suggesting that low *POSTN* levels may be associated with the anti-inflammatory and tissue repair functions of M2 macrophages, thereby reflecting the immune characteristics of chronic repair phases or low-inflammatory states in CAD. In contrast, in the high *POSTN* expression group, *POSTN* was negatively correlated with resting NK cells, indicating that high *POSTN* expression may be linked to the activation of inflammatory signaling pathways (such as NF-κB), thereby inhibiting the resting state of NK cells or altering their activity, which reflects differences in the immune microenvironment during acute or high-inflammatory states of CAD. These findings align with previous studies that have demonstrated the involvement of these immune cells in CAD pathogenesis (37, 38). Activated mast cells and neutrophils are known to contribute to inflammation and plaque instability, while Tregs play a protective role by modulating immune responses and maintaining vascular homeostasis (39–41).

Several limitations of this study must be acknowledged. First, the findings are derived solely from bioinformatics analyses, without integration with wet lab experiments, which could provide empirical validation. Second, the sample size of the datasets analyzed may limit the generalizability of the results. Additionally, the study lacks clinical validation, which is crucial for confirming the diagnostic and therapeutic relevance of the identified biomarkers and pathways. Last, the integration of multiple datasets, while beneficial for increasing statistical power, may introduce batch effects despite efforts to mitigate them, potentially affecting the robustness of the findings.

In conclusion, this study identified DEGs associated with CAD and elucidated their potential roles through functional enrichment and network analyses. The *POSTN* gene emerged as a significant marker with diagnostic potential, as supported by its expression profile and ROC curve analysis. These findings lay the groundwork for further research, including experimental validation and clinical trials, to explore therapeutic implications and improve the management of CAD.

## Data Availability

The raw data supporting the conclusions of this article will be made available by the authors, without undue reservation.
