# Supplementary material for: Serum periostin as a novel biomarker and therapeutic target in coronary heart disease
Source: Front Cardiovasc Med. 2025 Dec 2;12:1556634. doi: 10.3389/fcvm.2025.1556634 (PMC12705617; doi:10.3389/fcvm.2025.1556634)
Supplement: Supplementary file 1 [file Datasheet1.pdf]

Supplementary

Materials and Methods:

1.1 Data Download

Through the R package GEOquery[1] downloaded from GEO database[2] (https://www.ncbi.nlm.nih.gov/geo/), Coronary heart Disease (Coronary Artery diseases, CAD) GSE66360[3]GSE42148[4]The samples of dataset GSE66360 and GSE42148 were all from Homo sapiens, and the tissue source was Whole Blood. The specific information is shown in Table 1. Among them, the chip platform of dataset GSE66360 was GPL570, which included 49 coronary heart disease (CAD) samples and 50 Control samples. The chip platform of dataset GSE42148 was GPL13607, including 13 coronary artery disease (CAD) samples and 11 Control samples. All coronary artery disease (CAD) samples and Control samples were included in this study.

The R package sva[5] was used to debatch GSE66360 and GSE42148 to obtain the Combined GEO Datasets. The Combined Datasets included 62 coronary artery disease (CAD) samples and 61 Control samples. Finally, the R package limma[6] was used to standardize the Combined GEO Datasets, annotate probes, and normalize them. To verify the effect of removing batch effect, Principal Component Analysis (PCA)[7] is performed on the expression matrix after removing batch effect. PCA is a method of data dimensionality reduction, which extracts the feature vectors

(components) of data from high-dimensional data. To transform the data into low-dimensional data and display these features in 2D or 3D graphs.

1.2 Differentially expressed genes associated with coronary heart disease

According to the sample grouping of the Combined GEO Datasets, the samples were divided into coronary artery disease (CAD) group and Control (Control) group. The R package limma was used to perform differential analysis of genes in the coronary artery disease (CAD) group and the Control (Control) group. The threshold value of $|logFC| > 0.58$ and $adj.p< 0.05$ was set as the Differentially Expressed Genes (DEGs). Genes with $logFC > 0.58$ and $adj.p < 0.05$ were Up-regulated differentially expressed genes (degs). Genes with $logFC < -0.58$ and $adj.p < 0.05$ were Down-regulated genes. p value correction method was Benjamini-Hochberg (BH). The results of the difference analysis were plotted by volcano plot through the R package ggplot2. Subsequently, the expression value heatmap of differentially expressed genes (DEGs) was drawn by the R package pheatmap.

Next, coronary artery disease (CAD) samples from the Combined GEO Datasets were divided into High Expression group and Low Expression group according to the median expression value of POSTN gene. The R package limma was used to analyze the differential Expression of genes in the High Expression group and the Low Expression group in the CAD group. The threshold value of $|logFC| > 0.58$ and p value < 0.05 was set as the Differentially Expressed Genes (DEGs). Genes with $logFC > 0.58$ and p value < 0.05 were Up-regulated differentially expressed genes (degs).

Genes with logFC < -0.58 and p value < 0.05 were Down-regulated differentially expressed genes. The p value correction method was Benjamini-Hochberg (BH). The results of the difference analysis were plotted by volcano plot through the R package ggplot2. Subsequently, the expression value heatmap of differentially expressed genes (DEGs) was drawn by the R package pheatmap.

1.3 Differential expression verification and ROC curve analysis

In order to further explore the expression differences of gene POSTN between coronary artery disease (CAD) samples and Control samples in the Combined GEO Datasets, a group comparison map was drawn based on the expression level of gene POSTN. We then used R package pROC to plot the receiver operating characteristic (ROC) Curve of POSTN and calculate the Area Under the Curve (AUC) to evaluate the diagnostic effect of POSTN expression level on the occurrence of coronary artery disease (CAD). The area under the curve (AUC) of the ROC curve was generally between 0.5 and 1. The closer the AUC is to 1, the better the diagnostic performance. When AUC was between 0.5 and 0.7, the accuracy was low, when AUC was between 0.7 and 0.9, the accuracy was moderate, and when AUC was above 0.9, the accuracy was high.

1.4 Gene ontology (GO) and pathway (KEGG) enrichment analysis

Gene Ontology (GO) analysis[8] is a common method for large-scale functional enrichment studies, including Biological Process (BP), Cell Component (Cell Component), and biological process (BP). CC) and Molecular Function (MF). Kyoto Encyclopedia of Genes and Genomes

(KEGG)[9] is a widely used database storing information on genomes, biological pathways, diseases and drugs. We used R package clusterProfiler[10] to perform gene ontology (GO) and pathway (KEGG) enrichment analysis of differentially expressed genes (DEGs) from high and low expression group differential analysis in coronary artery disease (CAD) samples from integrated GEO Datasets (Combined Datasets). The entry screening criteria was adj.p < 0.05, and the p value correction method was Benjamini-Hochberg (BH).

1.5 Gene set Enrichment analysis (GSEA)

Gene Set Enrichment Analysis (GSEA)[11], known as GSEA, is used to evaluate the distribution trend of genes in a predefined gene set in a gene table ranked by their correlation with phenotype, and thus determine their contribution to phenotype. In this study, the genes of coronary artery disease (CAD) samples from the Combined GEO Datasets were first ranked according to logFC value. Then, the R package clusterProfiler was used to perform gene set enrichment analysis (GSEA) on all genes in the coronary heart disease (CAD) samples of the integrated GEO Datasets (Combined Datasets). The parameters used in GSEA were as follows: The seed is 2020, the number of computations is 1000, and the minimum number of genes contained in each gene set is 10 and the maximum number of genes contained in each gene set is 500. Through Molecular Signatures Database (MSigDB) Database[12] (https://www.gsea-msigdb.org/gsea/msigdb) access to c2 gene sets. Cp. All. V2022.1. Hs. Symbols. The GMT [All Canonical Pathways](3050) was used for gene set enrichment analysis (GSEA). The screening criteria of gene set enrichment

analysis (GSEA) were adj.p < 0.05 and FDR value (q value) < 0.25 were considered statistically significant. p value correction method was Benjamini-Hochberg (BH).

1.6 Gene Set Variation Analysis (GSVA)

Gene Set Variation Analysis (GSVA)[13], known as gene set variation analysis, is a nonparametric unsupervised analysis method that evaluates gene set enrichment results of microarray nuclear transcriptome by converting gene expression matrix between different samples into gene expression matrix between samples. To evaluate whether different pathways are enriched in different samples. Through Molecular Signatures Database (MSigDB) Database [12]access to c2. Cp. V2023.2. Hs. Symbols. The GMT gene set and using R package GSVA (Version 1.50.0) for integration of GEO data set (Combined Gene set variation analysis (GSVA) was performed on all genes of coronary artery disease (CAD) Datasets to calculate the difference in functional enrichment between the Low Expression group and the High Expression group of POSTN. The screening criterion for gene set variation analysis (GSVA) was a p-value < 0.05.

1.7 Protein-protein interaction (PPI) network

The GeneMANIA database[14] (https://genemania.org/) was used to generate hypotheses about gene function, analyze gene lists and prioritize genes for functional analysis. Given a list of query genes, GeneMANIA finds functionally similar genes using a large set of genomics and

proteomics data. In this mode, it weights each functional genomic dataset according to the predicted value of the query. Another use of GeneMANIA is gene function prediction. Given a query gene, GeneMANIA finds genes that are likely to share functions with it, based on how the gene interacts with it. A protein-protein interaction Network (PPI Network) was constructed by predicting functionally similar genes of gene POSTN through GeneMANIA online website.

1.8 Construction of regulatory network

Transcription factors (TFS) control gene expression by interacting with DDB2 at the post-transcriptional stage. By ChIPBase database[15] (http://rna.sysu.edu.cn/chipbase/) to retrieve the transcription factor (TF), analysis of transcription factor (TF) regulation, gene POSTN Cytoscape software was used to visualize the mRNA-TF Regulatory Network.

In addition, mirnas play an important regulatory role in the process of biological development and evolution. They can regulate a variety of target genes, and the same target gene can also be regulated by multiple mirnas. In order to analyze the relationship between the gene POSTN and micrornas, through a starBase[16] (https://rnasysu.com/encori/) access to related to gene POSTN micrornas, Cytoscape software was used to visualize the mRNA-miRNA Regulatory Network.

Finally, the Comparative Toxicogenomics Database[17] (https://ctdbase.org/) was used to predict the direct and indirect drug targets of gene POSTN,

and the interaction between gene POSTN and drugs was explored. Cytoscape software was used to visualize the mRNA-Drug Regulatory Network to complete the construction of the network.

1.9 Immune infiltration analysisCIBERSORT[18] is based on linear support vector regression to deconvolute the transcriptome expression matrix to estimate the composition and abundance of immune cells in a mixture of cells. The CIBERSORT algorithm, Combined with the LM22 feature gene matrix, and filtered out the data with immune cell enrichment score greater than zero, finally obtained the specific results of immune cell infiltration matrix in coronary artery disease (CAD) samples of GEO Datasets, and drew the proportion bar chart for display. Then, the correlation between immune cells in the high and low expression groups of POSTN was calculated based on Spearman algorithm, and the R package pheatmap was used to draw the correlation heatmap to show the correlation analysis results of immune cells themselves. The correlation between gene POSTN and immune cells was calculated based on Spearman algorithm, and the R package ggplot2 was used to draw a lollipop chart to show the correlation analysis results between gene POSTN and immune cells.

References:

[1] Davis S M P. GEOquery: a bridge between the Gene Expression Omnibus (GEO) and BioConductor [J]. Bioinformatics 2007 Jul 15;23(14):1846-7.

[2] Barrett T, Wilhite S E, Ledoux P, et al. NCBI GEO: archive for functional genomics data sets--update [J]. Nucleic acids research, 2013, 41(Database issue): D991-5.

[3] Carolan B J, Harvey B G, De B P, et al. Decreased expression of intelectin 1 in the human airway epithelium of smokers compared to nonsmokers [J]. J Immunol, 2008, 181(8): 5760-7.

[4] Shaykhiev R, Otaki F, Bonsu P, et al. Cigarette smoking reprograms apical junctional complex molecular architecture in the human airway epithelium in vivo [J]. Cell Mol Life Sci, 2011, 68(5): 877-92.
[5] Leek J T, Johnson W E, Parker H S, et al. The sva package for removing batch effects and other unwanted variation in high-throughput experiments [J]. Bioinformatics, 2012, 28(6): 882-3.
[6] Ritchie M E, Phipson B, Wu D, et al. limma powers differential expression analyses for RNA-sequencing and microarray studies [J]. Nucleic Acids Res, 2015, 43(7): e47.
[7] Ben Salem K, Ben Abdelaziz A. Principal Component Analysis (PCA) [J]. La Tunisie medicale, 2021, 99(4): 383-9.
[8] Mi H, Muruganujan A, Ebert D, et al. PANTHER version 14: more genomes, a new PANTHER GO-slim and improvements in enrichment analysis tools [J]. Nucleic Acids Res, 2019, 47(D1): D419-d26.
[9] Kanehisa M, Goto S. KEGG: kyoto encyclopedia of genes and genomes [J]. Nucleic Acids Res, 2000, 28(1): 27-30.
[10] Yu G, Wang L G, Han Y, et al. clusterProfiler: an R package for comparing biological themes among gene clusters [J]. Omics, 2012, 16(5): 284-7.
[11] Subramanian A, Tamayo P, Mootha V K, et al. Gene set enrichment analysis: a knowledge-based approach for interpreting genome-wide expression profiles [J]. Proc Natl Acad Sci U S A, 2005, 102(43): 15545-50.
[12] Liberzon A, Subramanian A, Pinchback R, et al. Molecular signatures database (MSigDB) 3.0 [J]. Bioinformatics, 2011, 27(12): 1739-40.
[13] Hanzelmann S, Castelo R, Guinney J. GSVA: gene set variation analysis for microarray and RNA-seq data [J]. BMC Bioinformatics, 2013, 14: 7.
[14] Franz M, Rodriguez H, Lopes C, et al. GeneMANIA update 2018 [J]. Nucleic acids research, 2018, 46(W1): W60-w4.
[15] Zhou K R, Liu S, Sun W J, et al. ChIPBase v2.0: decoding transcriptional regulatory networks of non-coding RNAs and protein-coding genes from ChIP-seq data [J]. Nucleic Acids Res, 2017, 45(D1): D43-d50.
[16] Vlachos I S, Paraskevopoulou M D, Karagkouni D, et al. DIANA-TarBase v7.0: indexing more than half a million experimentally supported miRNA:mRNA interactions [J]. Nucleic acids research, 2015, 43(Database issue): D153-9.
[17] Grondin C J, Davis A P, Wiegers J A, et al. Predicting molecular mechanisms, pathways, and health outcomes induced by Juul e-cigarette aerosol chemicals using the Comparative Toxicogenomics Database [J]. Curr Res Toxicol, 2021, 2: 272-81.
[18] Newman A M, Liu C L, Green M R, et al. Robust enumeration of cell subsets from tissue expression profiles [J]. Nat Methods, 2015, 12(5): 453-7.
[19] Bai X, Chen H, Oliver B G. miRNAs-mediated overexpression of Periostin is correlated with poor prognosis and immune infiltration in lung squamous cell carcinoma [J]. Aging (Albany NY), 2022, 14(9): 3757-81.
[20] Su Q, Deng Z, Wei X, et al. Serum Periostin as a Novel Biomarker for Predicting 30-Day Major Adverse Cardiac Events After Off-Pump Coronary Artery Bypass

Grafting [J]. Ther Clin Risk Manag, 2025, 21: 161-76.

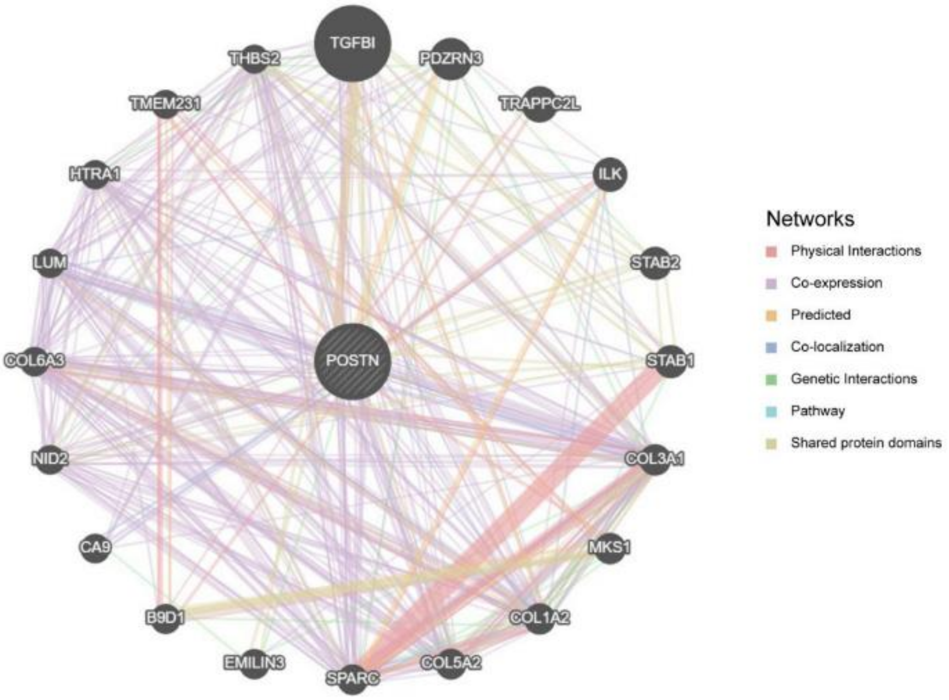

Supplementary Fig. 1 Protein-protein interaction network. Reproduced from "Analysis of neighboring gene networks in lung cancer. The gene-gene interaction network of POSTN constructed using GeneMania (A)" by Xu Bai, Hui Chen and Brian

G. Oliver, licensed under CC BY 3.0.

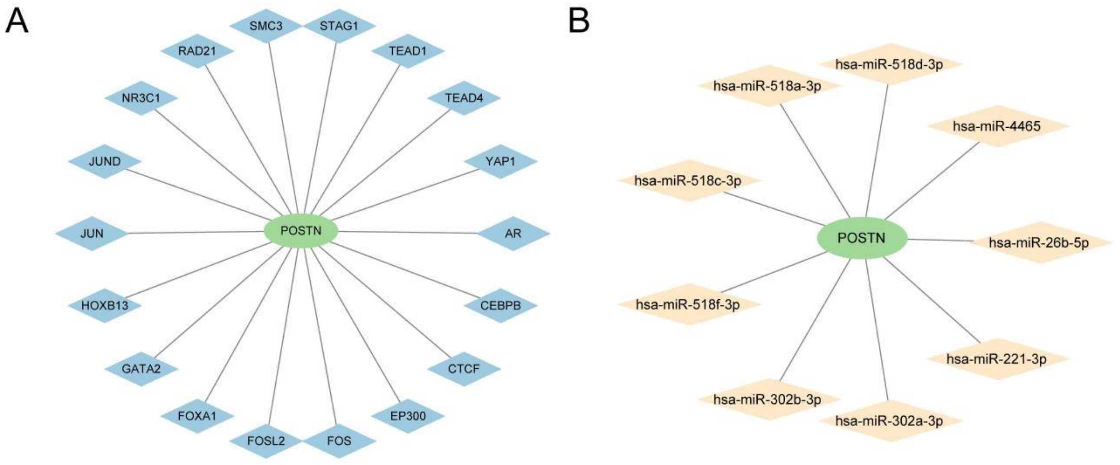

Supplementary Fig.2 Regulatory Network of POSTN

The mRNA-TF Regulatory Network of gene POSTN. B. mRNA-miRNA Regulatory Network of gene POSTN. mRNA: Messenger RNA , TF, Transcription Factor. Messenger RNA(mRNA) are shown in green, transcription factors (TFS) in blue, miRNAs in yellow.

Supplementary Table 1 **GEO Microarray Chip Information**[20]

	GSE66360	GSE42148
Platform	GPL570	GPL13607
Species	Homo sapiens	Homo sapiens
Tissue	Whole Blood	Whole Blood
Samples in CAD group	49	13
Samples in Control group	50	11
Reference	PMID: 28947747	PMID: 38573982

GEO, Gene Expression Omnibus; CAD, Coronary Artery Disease.

Supplementary Table 2 **Result of GO and KEGG Enrichment Analysis for DEGs**

ONTOLOGY	ID	Description	GeneRatio	BgRatio	pvalue	p.adjust	qvalue
BP	GO:1901889	negative regulation of cell junction assembly	6/186	32/18800	6.33E-07	1.30E-03	1.03E-03
BP	GO:1901888	regulation of cell junction assembly	12/186	201/18800	7.88E-07	1.30E-03	1.03E-03
BP	GO:0042060	wound healing	17/186	429/18800	1.33E-06	1.47E-03	1.16E-03
BP	GO:0043405	regulation of MAP kinase activity	11/186	183/18800	2.15E-06	1.78E-03	1.41E-03
BP	GO:0048661	positive regulation of smooth muscle cell proliferation	8/186	95/18800	4.61E-06	3.04E-03	2.41E-03

CC	GO:0062023	collagen-containing extracellular matrix	25/199	429/19594	1.93E-12	5.81E-10	5.24E-10
CC	GO:0005788	endoplasmic reticulum lumen	16/199	311/19594	1.26E-07	1.89E-05	1.71E-05
CC	GO:0031091	platelet alpha granule	8/199	91/19594	4.06E-06	3.92E-04	3.54E-04
CC	GO:0031093	platelet alpha granule lumen	7/199	67/19594	5.21E-06	3.92E-04	3.54E-04
CC	GO:0070820	tertiary granule	8/199	164/19594	2.78E-04	1.67E-02	1.51E-02
MF	GO:0005201	extracellular matrix structural constituent	17/192	172/18410	3.06E-12	1.26E-09	1.18E-09
MF	GO:000551	collagen binding	8/192	68/18410	5.24E-0	1.08E-0	1.01E-0

	8				7	4	4
	GO:001983			139/1841	1.54E-0	1.64E-0	1.53E-0
MF	8	growth factor binding	9/192	0	5	3	3
	GO:000196				1.60E-0	1.64E-0	1.53E-0
MF	8	fibronectin binding	5/192	31/18410	5	3	3
	GO:004801			489/1841	5.65E-0	4.59E-0	4.28E-0
MF	8	receptor ligand activity	16/192	0	5	3	3
					2.83E-0	5.69E-0	5.04E-0
KEGG	hsa05205	Proteoglycans in cancer	12/92	205/8164	6	4	4
					5.88E-0	5.91E-0	5.23E-0
KEGG	hsa04512	ECM-receptor interaction	8/92	88/8164	6	4	4
					3.49E-0	2.34E-0	2.07E-0
KEGG	hsa04668	TNF signaling pathway	8/92	112/8164	5	3	3

KEGG	hsa04933	AGE-RAGE signaling pathway in diabetic complications	7/92	100/8164	1.25E-04	6.30E-03	5.57E-03
KEGG	hsa05418	Fluid shear stress and atherosclerosis	8/92	139/8164	1.61E-04	6.48E-03	5.74E-03

GO, Gene Ontology; BP, Biological Process; CC, Cellular Component; MF, Molecular Function; KEGG, Kyoto Encyclopedia of Genes and Genomes; DEGs, Differentially Expressed Genes.

Supplementary Table 3 **Results of GSEA for Expression Group[20]**

ID	setSize	enrichmentScore	NES	pvalue	p.adjust	qvalue
REACTOME_REGULATION_OF_INSULIN_LIKE_GROWTH_FACTOR_IGF_TRANSPORT_AND_UPTAKE_BY_INSULIN_LIKE_GROWTH_FACTOR_BINDING_PROTEINS_IGFBPS	117	0.58	2.42	1.00E-10	1.23E-07	1.09E-07
REACTOME_ECM_PROTEOGLYCANS	73	0.60	2.34	2.94E-08	1.21E-05	1.07E-05
REACTOME_SYNDECAN_INTERACTIONS	25	0.76	2.33	1.38E-06	3.09E-04	2.73E-04
NABA_CORE_MATRISOME	248	0.50	2.33	1.00E-10	1.23E-07	1.09E-07
PID_INTEGRIN1_PATHWAY	65	0.61	2.33	7.66E-08	2.69E-0	2.38E-0

					5	5
WP_MIR5093P_ALTERATION_OF_YAP1ECM_AXIS	17	0.82	2.30	1.87E-06	3.83E-04	3.39E-04
WP_MIRNA_TARGETS_IN_ECM_AND_MEMBRANE_RECEPTORS	21	0.76	2.29	4.14E-06	6.78E-04	5.99E-04
REACTOME_NON_INTEGRIN_MEMBRANE_ECM_INTERACTIONS	56	0.61	2.26	7.18E-07	1.85E-04	1.63E-04
WP_APOPTOSISRELATED_NETWORK_DUE_TO_ALTERED_NOTCH3_IN_OVARIAN_CANCER	50	0.60	2.19	3.20E-06	5.62E-04	4.97E-04
WP_PHOTODYNAMIC_THERAPYINDUCED_NF-KB_SURVIVAL_SIGNALING	34	0.65	2.19	1.01E-05	1.46E-03	1.29E-03
PID_FRA_PATHWAY	34	0.65	2.18	1.24E-05	1.69E-03	1.49E-03

WP_PHOTODYNAMIC_THERAPYINDUCED_HIF1_SURVIVAL_SIGNALING	36	0.64	2.16	1.79E-05	2.09E-03	1.85E-03
NABA_ECM_GLYCOPROTEINS	171	0.49	2.15	2.53E-09	1.55E-06	1.37E-06
KEGG_ECM_RECEPTOR_INTERACTION	81	0.55	2.14	7.51E-07	1.85E-04	1.63E-04
REACTOME_INTERLEUKIN_4_AND_INTERLEUKIN_13_SIGNALING	104	0.52	2.13	3.49E-07	1.07E-04	9.49E-05
WP_DEVELOPMENT_AND_HETEROGENEITY_OF_THE_ILC_FAMILY	32	0.60	1.99	2.34E-04	1.20E-02	1.06E-02
BIOCARTA_IL1R_PATHWAY	28	0.60	1.89	1.18E-03	3.10E-02	2.74E-02
WP_SIGNAL_TRANSDUCTION_THROUGH_IL1R	30	0.58	1.87	8.39E-04	2.46E-0	2.17E-0

					2	2
					1.79E-0	1.59E-0
KEGG_TGF_BETA_SIGNALING_PATHWAY	81	0.46	1.79	4.66E-04	2	2
					2.58E-0	2.28E-0
WP_IL18_SIGNALING_PATHWAY	255	0.37	1.74	2.62E-05	3	3

GSEA, Gene Set Enrichment Analysis.

Supplementary Table 4 **Results of GSVA for Expression Group[20]**

Pathway	logFC	AveExpr	P.Value	adj.P.Val
HALLMARK_GLYCOLYSIS	-0.11679	0.00104	0.022617	0.16155
HALLMARK_CHOLESTEROL_HOMEOSTASIS	-0.13477	0.002503	0.035593	0.222459
HALLMARK_TGF_BETA_SIGNALING	-0.15297	0.011832	0.017472	0.148925
HALLMARK_ANDROGEN_RESPONSE	-0.15922	-0.00668	0.006169	0.077108
HALLMARK_ESTROGEN_RESPONSE_LATE	-0.17418	-0.02115	0.001592	0.02654
HALLMARK_ANGIOGENESIS	-0.18284	-0.01096	0.017871	0.148925
HALLMARK_UV_RESPONSE_DN	-0.18399	0.028455	0.001121	0.02654
HALLMARK_EPITHELIAL_MESENCHYMAL_TRANSITION	-0.22064	-0.0057	0.000291	0.014563

GSVA, Gene Set Variation A

Supplementary Table 5 Molecular Information

Pubmed id	Molecule Name	Structure	Molecular Weight	Vina Score
CID864	alpha-Lipoic acid	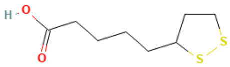	206.3 g/mol	-5.0 Kcal/mol
CID5351619	Erythrothioneine	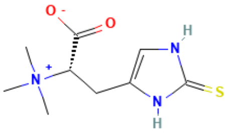	229.30 g/mol	-5.2 Kcal/mol
CID5743	Dexamethasone	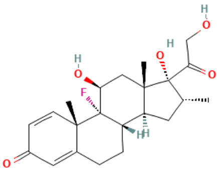	392.5 g/mol	-8.2 Kcal/mol
CID5206	Sevoflurane	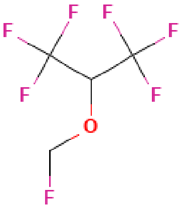	200.05 g/mol	-5.0 Kcal/mol
CID439153	NADH	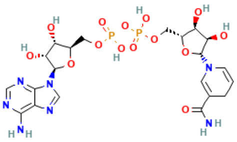	665.4 g/mol	-9.4 Kcal/mol
